# *Lycium barbarum* polysaccharide attenuates *Pseudomonas-aeruginosa* pyocyanin-induced cellular injury in mice airway epithelial cells

**DOI:** 10.29219/fnr.v66.4585

**Published:** 2022-02-14

**Authors:** Xue Lin, Fuyang Song, Yiming Wu, Di Xue, Yujiong Wang

**Affiliations:** 1 Key Laboratory of Ministry of Education for Conservation and Utilization of Special Biological Resources in the Western, Ningxia University, Yinchuan, Ningxia, China; 2 College of Life Science, Ningxia University, Yinchuan, Ningxia, China

**Keywords:** *Lycium barbarum* polysaccharide (LBP), pyocyanin (PCN), air-liquid interface, cell injury

## Abstract

**Background:**

*Lycium barbarum* berries have been utilized in Asia for many years. However, the mechanisms of its lung-defensive properties are indeterminate.

**Objective:**

We investigate whether *L. barbarum* polysaccharide (LBP) could weaken *Pseudomonas aeruginosa* infection-induced lung injury.

**Design:**

Mice primary air-liquid interface epithelial cultures were pretreated with LBP and subsequently treated with pyocyanin (PCN). Lung injury, including apoptosis, inflammation, and oxidative stress, was estimated by western blot, enzyme-linked immunosorbent assay, *and real-time quantitative polymerase chain reaction, Real-time qPCR (Q-PCR)*. Flow cytometry was used to test cell apoptosis. Moreover, Balb/c mice were used to evaluate the tissue injury. We used hematoxylin-eosin staining and immunofluorescence to detect the expression of related proteins and tissue damage in mouse lungs and spleen.

**Results:**

The flow cytometric analysis shows the potential of LBP to reduce time-dependent cell death by PCN. Mechanistically, LBP reduces PCN-induced expression of proapoptotic proteins and caspase3 and induces the activation of Bcl-2 in mice bronchial epithelial cells. Similarly, LBP reduces PCN-induced intracellular reactive oxygen species (ROS) production. Moreover, LBP inhibits the production of inflammatory cytokines, Interleukin (IL-1β), Tumor Necrosis Factor (*TNF)*, IL-6, and IL-8. Our study confirms the ability of LBP to retard PCN-induced injury in mice lung and spleen.

**Conclusions:**

The inhibition of PCN-induced lung injury by LBP is capable of protecting mice cells from injury.

## Popular scientific summary

- Investigating the effects of *L. barbarum* polysaccharide on pyocyanin infection.
- Using air-liquid culture as an infection model.
- Indicating the potential applicability of LBP as an antimicrobial agent.

*Lycium barbarum* (goqi or wolfberry), a member of the eggplant family, is a fruit that has long been used as tea and herb in China and as a dietary supplement for functional food ingredients or micronutrients (1). Several plant chemical substances, such as flavonoids, phenolic compounds, terpene, saponins, and polysaccharides, can be separated from their fruits (1, 2). *L. barbarum* polysaccharide (LBP) is the main active ingredient of *L. barbarum*, which has many biological activities, such as antioxidant and hypoglycemic effects and anticancer activity (2–4). However, there is limited information on the mechanisms of immunomodulatory activities stimulated by LBP (5). *Pseudomonas aeruginosa* is a long gram-negative bacillus that is aerobic and has unilateral mobility. It secretes various pigments, including pyocyanine, luciferin, and pyocyanin (6). Studies have shown that pyocyanin (PCN) is an important causative agent of *P. aeruginosa*. PCN can cause leukopenia, tissue necrosis, and organ failure in humans (7, 8). As the infection time prolongs, PCN can cause death. PCN has three functional regions: the receptor-binding region, the transmembrane transport region, and the toxic region (i.e. the Adenosine diphosphate [*ADP]* ribosylase region), which bind to the cell’s receptors surface by receptor-binding regions (9–11).

Results of previous research have demonstrated the potential of LBP to suppress endoplasmic reticulum stress (ERS), which reduces apoptosis (11). Furthermore, LBP potentially improves lipid profiles and oxygen status and reduces abdominal fat in patients with metabolic syndrome. However, the effects of LBP on inhibiting bacterial growth and preventing biological infections remain unclear. Studies have shown that PCN can reduce the bronchial mucus flow rate, conducive to *P. aeruginosa* colonization of the respiratory tract (12). At the same time, PCN can also reduce the respiratory tract’s cilia swaying speed and finally stop the respiratory tract’s cilia from oscillating, leading to damage of the epithelial cells (7, 13). *In vitro* studies have shown that PCN affects many cell’s functions, but its preclinical infections’ specific mechanism is still unclear. However, despite the extensive scientific discussion of *P. aeruginosa* virulence factors in the literature, the organism’s mechanisms of evading macrophage resistance are indistinct.

In this study, we generated an air-liquid interface (ALI) culture system of mouse bronchial epithelial cells to analyze their morphological composition. We used the ALI culture system to explore further the immunological response and host cells’ damage after *P. aeruginosa* infection. We aim to investigate the potential of LBP pretreatment in alleviating *P. aeruginosa*. Animal experiments were used to elicit the role of LBP in antagonizing *P. aeruginosa* infection. Our study scientifically proved that LBP is the main driver of the immunity function of *Lycium barbarum*.

## Method

### Materials

LBP of 95% purity was purchased from Shanghai Biological Co. The LBP was dissolved in ultrapure water and stored at 4°C. All analytical reagents were used directly without further purification. Fresh stock solutions were prepared after 15 days.

### Cell culture

All animal experimental procedures were performed at Ningxia University. This study was approved by the animal ethics committee of Ningxia University with the approval number 2018-019. Sixty (60) Balb/c mice (6 weeks old female) were purchased from the Shanghai Laboratory Animal Center of the Chinese Academy of Sciences. The animals were housed in a controlled temperature environment. We developed the mice airway epithelia’s primary culture at the ALI (12, 13). Ten percent (10%) fetal bovine serum (FBS) was added to the culture and finally converged to terminate enzymatic dissociation. Subsequently, the culture was centrifuged for 10 min at 4°C to collect 500 g epithelial cells. The cells were resuspended in Dulbecco’s Modified Eagle Medium (DMEM) 10% *FBS* culture, and the cells on the tissue culture plate incubate the fibroblasts in 5% CO_2_ for 2–4 h at 37°C. A 500 g of non-disciple cells was centrifuged at 4°C for 10 min, and the cells in the bronchial epithelial cell growth medium were collected. We used an automated vision-based cell counter to determine the total number of cells. *P. aeruginosa* was used to infect the 6-week ALI cultures. Cells were infected on the culture model’s apical surface for 20 min at a 30-fold multiplicity infection rate. Culture medium containing uncultured *P. aeruginosa* was removed from the top of the culture. *P. aeruginosa* was cocultured with epithelial cells for 36 h, and the cells were collected after coculture. The morphology of the cells and bacterial colonization were observed by hematoxylin-eosin staining. All staining reagents were obtained from Sigma-Aldrich (Missouri, USA). We observed mice bronchial epithelial cells’ characteristics by utilizing immunofluorescent staining of tubulin4 and keratin14. PAS staining was used to detect glycogen secretion in cells. Tubulin 4, keratin 14, and PAS staining: Periodic Acid-Schiff stain (PAS) staining were assessed in mice bronchial epithelial cells.

### Identification of cells surface marking molecules and immunofluorescent staining

The cells were fixed with 4% paraformaldehyde for 15 min at room temperature. The fixed cells were washed with PBS three times and then permeabilized with 0.3% Triton X-100 for 20 min. Furthermore, the cells were blocked in 5% ordinary goat serum for 1–2 h at room temperature. Rabbit antikeratin 14 and PAS antibodies (Thermo, Rockford, USA), and rabbit anti-*P. aeruginosa* (home-made antibody; 1:200) were added to the cells and incubated overnight at 4°C. After removing the primary antibody, the cells were washed three times using PBS. Alexa Fluor 594-labeled antibody (green) and Alexa Fluor 594-labeled antibody (red; Jackson Immuno Research Laboratories, West Grove, PA, USA; 1:500) were added to the cells as fluorescent secondary antibodies to detect primary antibodies. The fluorescent secondary antibodies were removed and washed three times with PBS, and the membrane was fixed on a glass slide by Vectashield Mounting Medium (H-1200, Vector Laboratories, Burlingame, CA) with 4’ 6-diamidino-2-phenylindole (DAPI). Colocalization images were acquired by the Leica TCS SP2 A0BS confocal system and analyzed by the Leica Confocal Software v. 2.6.1 (Leica, Germany).

### Cell viability assay

The ALI-cultured bronchial epithelial cells were plated in 96-well plates at a concentration of 5 × 10^3^ cells/well. ALI-cultured bronchial epithelial cells were incubated with 5% CO_2_ at 37°C for 2 h. The PCN-treated group was subsequently treated with a gradient concentration of PCN at the time points 6, 12, and 24 h. The LBP-pretreated group was pretreated with LBP at gradient concentration for 2 h before adding PCN. Cell activity was detected by the 3-(4,5)-dimethylthiahiazo (-z-y1)-3,5-di- phenytetrazoliumromide (*MTT)* method according to the kit instructions. Briefly, 20 μL MTT (5 mg/mL; Sigma-Aldrich, Saint Louis, MO) was added to the previous section’s processing groups. The MTT-added medium and the cells were incubated at 37°C for 4 h. The plate was read at 570 nm using a microplate reader (Model 680; Bio-Rad Laboratories Inc, Hercules, CA), according to the reference wavelength. Untreated cells were used as controls, and the wells with PBS served as blank control wells. The average optical density (OD) was subtracted from the OD of the sample compared with the control cells’ OD. We used the following formula to calculate the percentage of cell viability: average density (OD) of the treated group − blank/mean OD of control cells × 100%.

### Western blot

Cells were lysed in a cell lysis buffer to obtain the total cellular extracts. The Pierce bicinchoninic acid (*BCA)* Protein Assay Kit was employed to determine the content of total protein in lysis cellular extracts (14). The sample protein was separated using 10% sodium dodecyl sulfate polyacrylamide gel electrophoresis (SDS-PAGE), and BCA quantified the protein sample concentration. Finally, the amount used in 10% SDS-PAGE was 20 μg. A nitrocellulose membrane (Mini-PROTEAN Tetra Cell, Bio Rad, Hercules, CA) was used to blot the SDS-PAGE. The HRP-conjugated secondary Ab or FLA9000 (Fuji Film, Minato, Japan) protein was visualized by electrochemiluminescence (ECL) using ChemiDoc-It (UVP, Upland, CA). The strip densitometry was performed using ImageJ Freeware (http://rsbweb.nih.gov/ij/). Antibodies used for blotting were Bcl-xl, 1:1,000; Bcl-2, 1:500; caspase3, 1:1,000; caspase9, 1:500 (Proteintech Group, USA); cytochrome C, 1:1,000 (Abcam, UK); and PARP-1, 1:500 (Cell Signal, USA).

### Real-time quantitative polymerase chain reaction analysis

The total RNA from mouse bronchial epithelial cells was isolated using TRIzol^®^ reagent. Then, the sample was reverse transcribed from RNA to cDNA using a Prime Script RT kit. Polymerase chain reaction (PCR) amplification was performed on the ABI 7500 Fast Thermal Cycler (Applied Biosystems, USA), according to the SYBR Green PCR Kit (Takara Biotechnology Co., Ltd., Dalian, China). PCR cycle was conducted for 30 s at 95°C, followed by 40 cycles at 95°C for 5 s and an annealing/extension step at 60°C for 15 s. The primer was designed by the Shanghai Sango Company (Shanghai, China). The specific primers are shown in Table 1.

**Table1:** qRT-PCR primer sequences

Gene	Forward (5’-3’)	Reverse (5’-3’)
Caspase3	GGTGCCTATGTCTCAGCCTCTT	GCCATAGAACTGATGAGAGGGAG
p62	TGGACCTTCCAGGATGAGGACA	GTTCATCTCGGAGCCTGTAGTG
Beclin-1	TACCACTTCACAAGTCGGAGGC	CTGCAAGTGCATCATCGTTGTTC
PARP	GACAGCCTGTGTTCGAGGATATG	TGTTCTTACAGGAGAGGGTAGAC
BAD	CATCACTGCCACCCAGAAGACTG	ATGCCAGTGAGCTTCCCGTTCAG
mTOR	TGAAAACACAGAAGTAACGTCCG	CCCAGGAGGAAATTGTAATGGGA
TGF-β	CTGGACTCATCGCAAACACAA	AGGAAGCCTTTGACTTCTGTCTA
NF-κB	AGATACTGCAAAGGATGCTCAAA	CAGCCTGATGGAATCATGGTC
Bcl-2	CCCATCTTTGAGCATCTTGGT	GCCCAGCCTGAGTAGTGAAG
Bcl-xl	CATCTTGGTTTCAAGCCCAGA	CTGCCCAGGCCAAAATTGC
β-actin	TTTGTTACCAGGCTCTCTTCC	GAATTGGGGCTTAGGCATCCA

### Measurement of intracellular reactive oxygen species

The ALI-cultured bronchial epithelial cells were plated in 96-well plates at a concentration of 5 × 10^3^ cells/well. PCN (50 μM) was added to the bronchial epithelial cells and incubated with PCN at 5% CO_2_ atmosphere at 37°C for 24 h after LBP treatment for 2 h. Twenty-five micromolar DCFH-DA was added to the plate, and the treated cells were incubated with DCFH-DA at 37°C for 30 min. The fluorescence was measured using a Microplate Reader (BIO-TEK, INC). According to the kit instructions, excitation and emission were set to 485 and 535 nm, respectively. Immunofluorescence microscopy was used to capture the reactive oxygen species (ROS) reaction. The average level of ROS was measured using a ROS assay kit DCFH-DA (Beyotime Biotech, Nanjing, China). Approximately 3 × 10^5^ cells/well were seeded in 6-well plates overnight, followed by PCN treatment with or without LBP pretreatment for 24 h. DCFH-DA was diluted to a final concentration of 10 μM. The cells were collected and suspended in diluted DCFH-DA in the dark at 37°C for 30 min and washed three times with PBS. The resulting samples were analyzed using an Accuri C6 Flow Cytometer (BD Biosciences, Franklin Lakes, NJ, USA).

### Enzyme-linked immunosorbent assay

PCN (50 μM) was added to the mouse bronchial epithelial cells medium and incubated in a 5% CO_2_ atmosphere at 37°C for 24 h. The above-mentioned PCN concentration (50 μM) was added to the cell cultures. The cells were incubated in a 5% CO_2_ in the atmosphere at 37°C for 24 h after LBP treatment for 2 h. Subsequently, the culture supernatant for inflammatory factors detection was collected. According to the kit instructions, TNF, IL-6, and IL-8 concentrations in the cell supernatant were also determined by enzyme-linked immunosorbent assay (ELISA).

### Histopathology and immunohistochemistry evaluation

The animals were randomly divided into six groups (*n* = 9). Group I animals were set as the blank control group and treated with 50 μL of physiological saline. Group II animals were set as the PCN treatment group and treated with 50 μg/50 μL PCN. Group III animals were set as the LBP treatment group and treated with LBP at a dosage of 3 mg/kg. Group IV animals were set as the *P. aeruginosa* treatment group and treated with plaque forming unit (*PFU)* of 1 × 10^6^ colony forming unit (CFU). Group V animals were set as the PCN treatment group after LBP pretreatment, and the treatment conditions were as described above. The Group VI animals were set as the *P. aeruginosa* treatment group after LBP pretreatment, and the treatment conditions were as described above. All the above treatments were imposed on the mice by intranasal route for 7 days. According to the aforementioned groups, for 7 days, the mice were sacrificed by cervical dislocation after treatment imposition. The mice were dissected after simple disinfection, and the lung and spleen tissues were collected for further morphological analysis. The lung and spleen sections were blocked at room temperature using saline containing 0.1% bovine serum albumin (*BSA)*. The sections were gently incubated for 2 h using the following primary antibodies: Bcl-2 (Abcam), Caspase-3 (NIMP-R14, Abcam), and Cyt-c (NB600-404, Novus Biologicals). Furthermore, we removed the primary antibodies, thoroughly washed the membranes with 0.5% TBS-Tween 20, and subsequently incubated and shook them gently for 2 h after adding a biotinylated secondary antibody. At least five slices per organism were manufactured for each treatment group. We captured images at 200× or 400× using the Zeiss microscope and ZEN software.

### Statistical analysis

All data collected were obtained from at least three independent experiments for each condition. The GraphPad Prism version 7.0 (GraphPad Prism Software Inc., La Jolla) was used for data analysis and graphic images. Statistical evaluation of the data was performed using a *t*-test to compare differences between the treatment groups. The difference was statistically significant (*P < 0.05; **P < 0.01; ***P < 0.001).

## Results

### Identification of ALI mice bronchial epithelial cells surface marking molecules and P. aeruginosa binding site

**Fig. 1:**
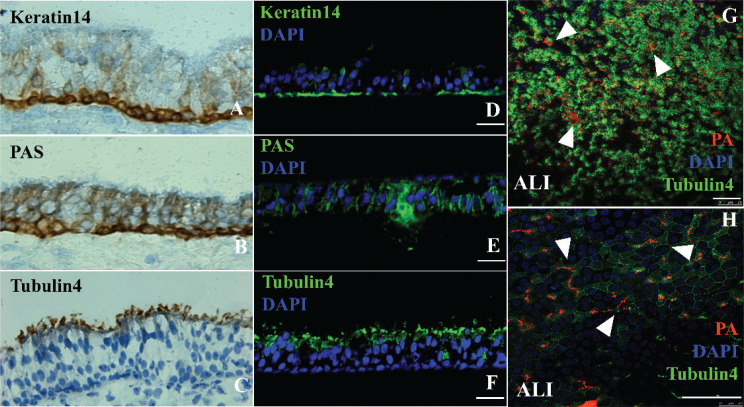
The differentiation of epithelial cells was ascertained by HE stain and immunofluorescent staining with specific markers for indicated epithelial cell types (a–f). *P. aeruginosa* binds to ciliated cells of primary of ALI sheep bronchial epithelia *in vitro* (d–h). (a, d) HE stain and immunostaining show the basal cells with the expression of cytokeratin 14 (K14) in ALI culture; (b, e) HE stain and immunostaining show the secretory cells with periodic acid-Schiff (PAS) staining in ALI culture; (c, f) HE stain and immunostaining show the ciliary axonemes with the expression of tubulin4 in ALI culture; (g, h) a representative image of confocal microscopy for ALI culture after binding of *P. aeruginosa* for 20 min and cultured for additional 24 h. Bars in a–h: 50 μm.

To characterize the differentiation of *in vitro* bronchial epithelial cells in mice, we identified the epithelial cell type by keratins4 and tubing4. PAS staining was used to detect glycogen secretion. The expression profile of the site depends on the differentiation level. Similar to the natural mouse bronchial tree, mouse bronchial epithelial cultures showed keratin 14 expression only in the PAS (characteristic features of the remnant cells) of the basal layer (Fig. 1a and d), secretory cells (Fig. 1b and e), and tubulin4 (Fig. 1c and f) in ciliary axonemes (a characteristic feature of ciliated cells). Previous studies have shown that *P. aeruginosa* mainly colonizes both ciliated and nonciliated cells. Consistent with these findings, we conducted confocal microscopy of *P. aeruginosa*, which binds early to populate related epithelial cell cultures rapidly. However, *P. aeruginosa* cells were more uniformly distributed on the epithelial surface (Fig. 1g and h). The morphological and immunochemical data strongly suggest the successful establishment of highly differentiated bronchial epithelial cells of the mice *in vitro*.

### LBP attenuate the loss of epithelial cell viability induced by PCN

**Fig. 2:**
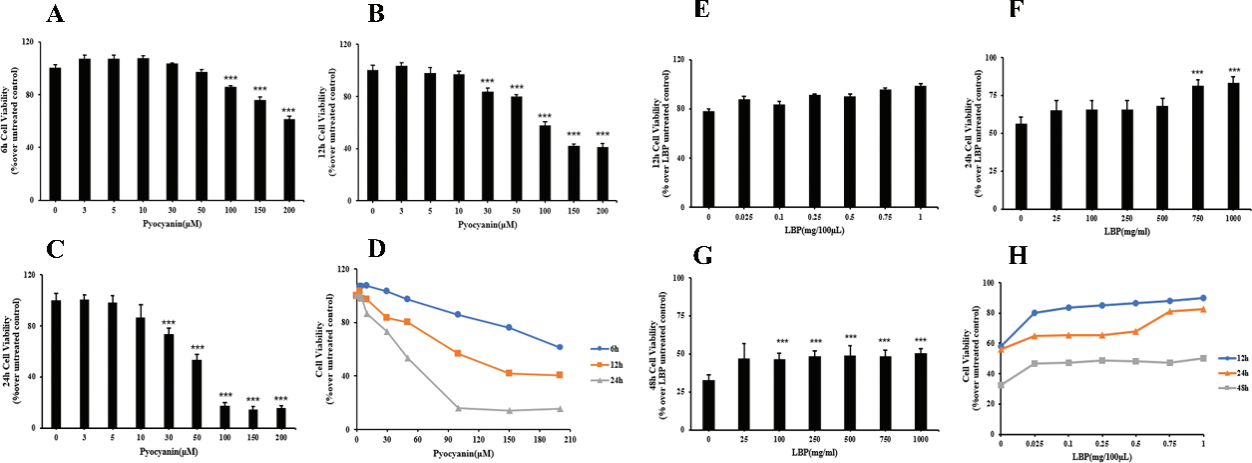
Sensitivity of PCN to treatment with the indicated doses of LBP as assessed by MTT assay. Pyocyanin reduced cell viability time- and concentration-dependent. Panels show dose-response viability curves to the indicated treatments. (a–d) The cell viability of epithelial cells after infected with PCN at the concentration of 0–100 μM for 6, 12, and 24 h. (e–h) The effects of LBP on epithelial cells viability following PCN insult, the cells were exposed to 50 μM PCN for 12, 24, and 48 h after incubation with 0–1,000 mg/mL LBP for 2 h. Data were presented as mean ± SD, compared with uninfected controls; *P < 0.05, **P < 0.01, and ***P < 0.001.

Cell activity was detected using the MTT method to reflect the cytotoxicity of PCN. Cells were incubated for 6, 12, and 24 h with 0–200 μM PCN before further testing. As a result, PCN’s dose-dependent cytotoxicity was evident to macrophage epithelial cells (Fig. 2d). We found a significant reduction of cell viability in epithelial cells exposed to PCN at 100 μM compared to the untreated controls (*P* < 0.001) after a 6 h infection (Fig. 2a). At 24 and 48 h, cell survival significantly reduced at 30 μM compared to the untreated control (*P* < 0.001; Fig. 2b and c). Therefore, a concentration of 50 μM was used in subsequent studies in this report. To investigate the effect of LBP on the survival of epithelial cells following PCN injury, 0–1,000 mg/mL LBP was cultured and exposed to 50 μM PCN. LBP-pretreated cells showed a significantly higher survival rate, while PCN damage significantly reduced the cell’s viability (Fig. 2e–h).

### LBP inhibits the inflammatory activation of a series of cytokines after PCN infection

**Fig. 3:**
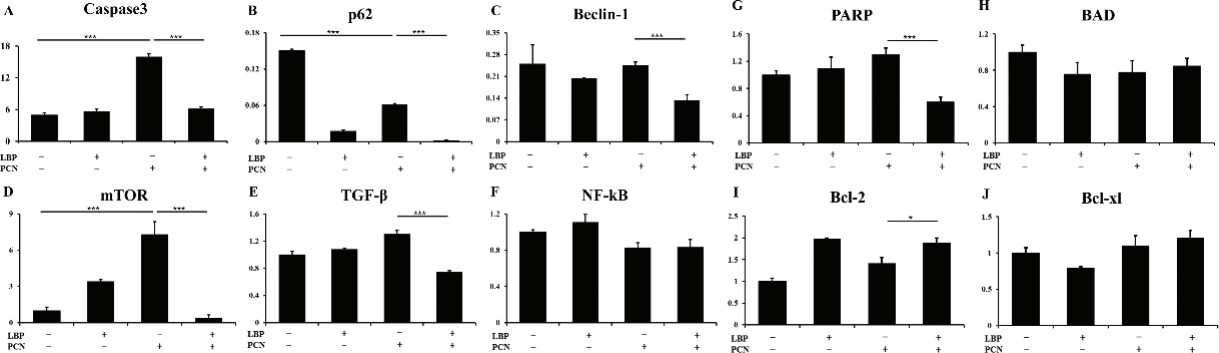
‘LBP inhibited a series of pro-apoptosis cytokines activation after PCN infection. Effects of LBP on 50 μM PCN-induced activation of cytokines activation were measured by q-PCR. Change of secreted cytokines content of (a) caspase3, (b) p62, (c) beclin-1, (d) PARP, (e) BAD, (f) mTOR, (g) TGF-β, (h) NF-κB, (i) Bcl-2, and (j) Bcl-xl. Data were presented as mean ± SD, compared with the uninfected controls, *P < 0.05, **P < 0.01 (*N* = 9 from three independent experiments).

Caspase3, p62, Beclin-1, PARP, BAD, mTOR, TGF-β, NF-κB, Bcl-2, and Bcl-xl are cytokines that are important for apoptosis control. The expression of the cytokines Caspase3, p62, Beclin-1, PARP, BAD, mTOR, TGF-β, and NF-κB was induced in cells infected with PCN infection compared to the uninfected control group (Fig. 3a–f, *P* < 0.001). The expression of Bcl-2 and Bcl-xl decreased in PCN-infected cells (Fig. 3i and j) compared to the uninfected control group, and the expression of Bcl-2 and Bcl-xl (Fig. 3i and j, *P* < 0.05) increased in the LBP pretreated group. LBP could also decrease the expression of pro-apoptosis cytokines. The expression of the cytokines Caspase3, p62, Beclin-1, PARP, BAD, mTOR, TGF-β, and NF-κB was decreased in cells pretreated with LBP compared to the LBP untreated PCN infection group (Fig. 3a–f, *P* < 0.001).

### LBP inhibits cell apoptosis induced by PCN in vitro

**Fig. 4:**
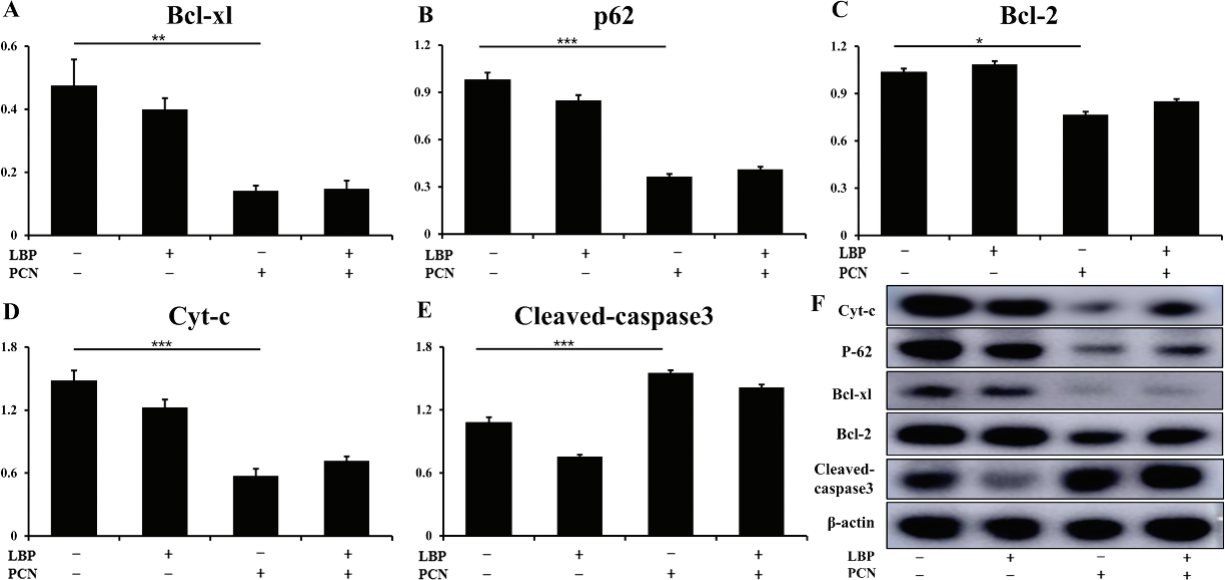
The cell apoptosis induced by PCN infection was pretreated with or without LBP for 2 h. Then, cells were infected with PCN at a concentration of 50 μM for 24 h. An involvement of Bcl-2/Bcl-xl protein in epithelial cells in response to PCN infection was pretreated with or without LBP for 2 h. Then, cells were infected with PCN at a concentration of 50 μM for 24 h. (f) Representative immunoblots of indicated protein from three repeated experiments. (a–e) Semiquantitative the expression of indicated proteins byoptical densitometry assay using ImageJ Software version 1.46. Data were presented as mean ± SD, compared with uninfected controls, *P < 0.05; **P < 0.01; ***P < 0.001.

Our result confirmed that LBP could inhibit apoptosis in A549 and PC12 cells, but it did not affect epithelial cells. Western blot also demonstrated that LBP (*P* < 0.05) suppressed the expression of active caspase-3 (Fig. 4e) compared to the PCN arbitration group. However, the Bcl-xl (Fig. 4c), cytochrome c release (Fig. 4d), and Bcl-2 (Fig. 4a) expressions had no statistical difference. These results suggest that LBP plays a protective role in epithelial cells, partly by antiapoptotic properties.

### LBP inhibits the production of epithelial cells intracellular ROS induced by PCN in vitro

**Fig. 5:**
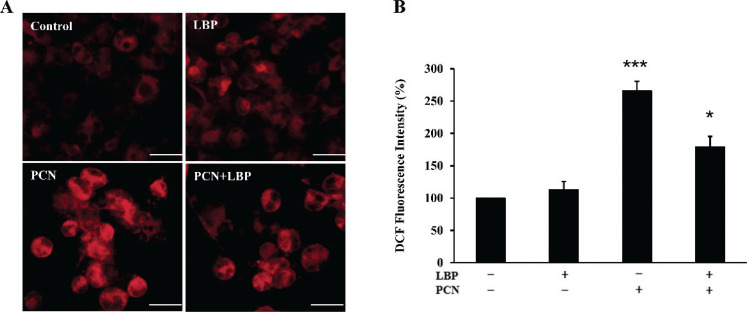
The concentration of ROS induced by PCN infection was pretreated with or without LBP for 2 h. Then, cells were infected with PCN at a concentration of 50 μM for 24 h to detect the expression of ROS. (a) Images were photographed using the confocal fluorescence microscope to detect the expression of ROS. (b) A microplate reader was used to quantify the ROS levels. Data were presented as mean ± SD, compared with uninfected controls, *P < 0.05; ***P < 0.001.

ROS is an important contributor to the development of cell apoptosis. The fluorescence intensity reflected the accumulated level of ROS. The fluorescence intensity of ROS was determined using a fluorescence microplate reader. We investigated epithelial cells’ intracellular ROS production induced by PCN and the suppression effect of LBP by measuring fluorescence intensity (Fig. 5b). Meanwhile, MitoSOX Red, a mitochondrial superoxide indicator, detected elevated mitochondrial ROS levels in PCN-treated cells (Fig. 5a). In LBP-pretreated cells, the fluorescence intensity becomes weak or disappears. The PCN apparently increased the ROS staining signal, while the LBP-pretreated group could reduce ROS expression induced by PCN.

### LBP inhibits epithelial cell ROS-dependent cell apoptosis induced by PCN in vitro

**Fig. 6:**
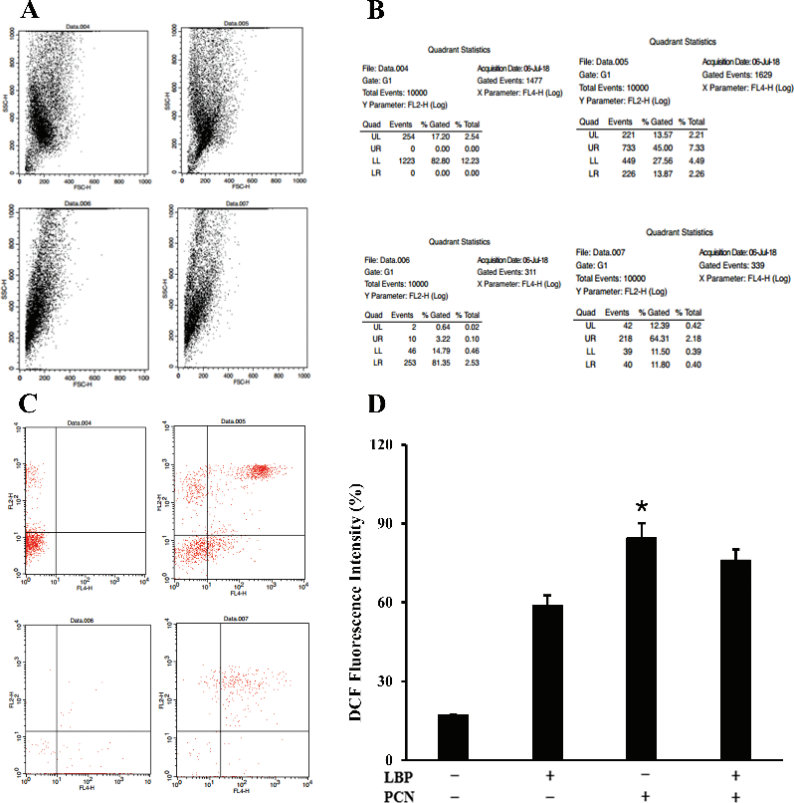
The cell apoptosis induced by PCN infection was pretreated with or without LBP for 2 h. Then, cells were infected with PCN at a concentration of 50 μM for 24 h. Subsequently, the cells were stained with annexin-V-FITC and were subsequently stained with PI in the dark. (a–c) Representative quadrant regions of flow cytometry analysis of indicated condition showing dot plot of each sub-population. (d) Quantitative data of cytometry analysis of indicated condition for different time points showing percentages of apoptotic cells. Data were presented as mean ± SD, compared with uninfected controls, *P < 0.05.

The percentage of apoptotic cells (annexin – V + PI + and annexin – V + PI -) was exposed to PCN treatment at a concentration of 50 μM at 84% and 57% on the treated cells for 24 h (Fig. 6d). Epithelial cells were pretreated with LBP before PCN treatment to investigate whether increased cell apoptosis was due to PCN infection and whether LBP could protect cells from this apoptosis. Results show that cell-induced PCN apoptosis may be inhibited by LBP (Fig. 6d, *P* < 0.05). The proportion of apoptotic cells was 76.11% in cells pretreated with LBP and exposed to PCN infection for 24 h at a concentration of 50 μM (Fig. 6d). These data suggest that LBP could inhibit PCN-induced apoptosis.

### LBP inhibits the production of PCN-induced inflammatory cytokines in vitro

**Fig. 7:**
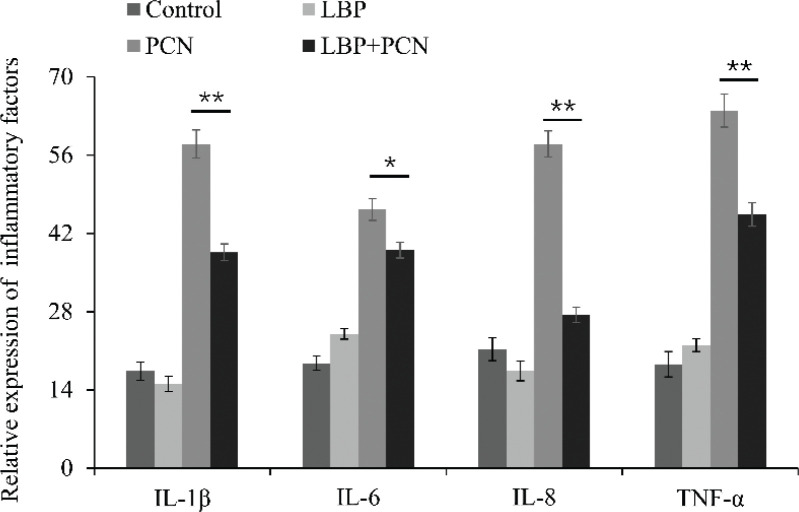
The concentration of IL-1β, TNF, IL-6, and IL-8 induced by PCN infection was pretreated with or without LBP for 2 h. Then, cells were infected with PCN at a concentration of 50 μM for 24 h to detect the expression. Data were presented as mean ± SD, compared with uninfected controls, *P < 0.05; **P < 0.01.

For the characterization of cytokine levels in PCN-induced cellular damage and LBP-mediated protection, IL-1β, TNF, IL-6, and IL-8 secreted protein levels were measured by ELISA in all groups at 24 h. PCN increases the secretion of IL-1β, TNF, IL-6, and IL-8 after 24 h treatment (Fig. 7). Particularly, LBP reversed such effects without affecting their basal levels (Fig. 7, *P* < 0.05, *P* < 0.01).

### LBP inhibits PCN-induced tissue apoptosis and injury in Balb/c mice

To elucidate the mechanism by which PCN and *P. aeruginosa* inhibit Bcl-2, Cytc, and Caspase-3, we used immunofluorescence of lung and spleen tissues to examine the interaction between LBP, PCN, and *P. aeruginosa*. The results indicate that the expression of Caspase-3 and cytochrome c increased under PCN and *P. aeruginosa* infection but decreased when pretreated with LBP.

**Fig. 8:**
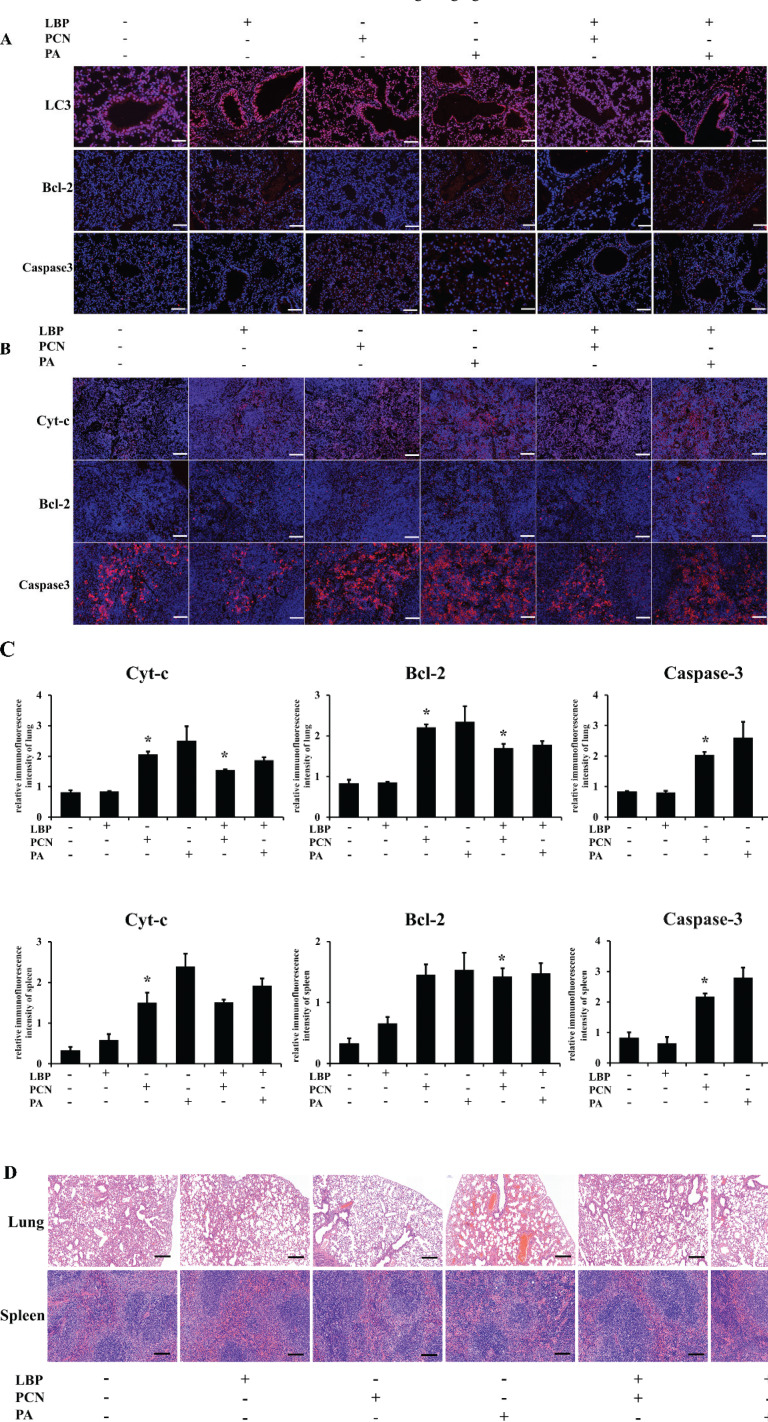
LBP contributes to enhance immune suppression during PCN and PA infection *in vivo*. Animals were randomly divided into five groups (*n* = 6). The Balb/c mice induced by PCN or PA infection were pretreated with or without LBP for 2 h. (a, b) Lung and spleen sections were stained with Bcl-2, Cyt-c, and caspase3 primary antibodies and visualized with a goat anti-Rabbit DyLightH 488-conjugated secondary antibody. Nuclei were stained with DAPI. Images were photographed using the confocal fluorescence microscope. (c) Hematoxylin and eosin (H&E) staining of the lungs and spleens of mice treated as above. Scale bars, 200 μm. (d) Representative immunoblots of Cyt-c, Bcl-2, and caspase3 from three repeated experiments. Data were presented as mean ± SD, compared with uninfected controls, *P < 0.05; **P < 0.01; ***P < 0.001.

As shown in Fig. 8a and b, lung and spleen immunofluorescence, respectively, show that LBP significantly suppressed the expression of active caspase3 (Fig. 8c) and inhibited cytochrome c release (Fig. 8c). These results suggest that LBP exerts a protective role on the tissues of the lungs and spleens of mice, at least partly through its antidepressant property. To confirm the immunity suppression of PCN and *P. aeruginosa* and LBP protection, we endotracheally induced Balb/c mice with LBP, PCN, and *P. aeruginosa*. The lung and spleen tissues in mice were analyzed by staining with hematoxylin-eosin. Normal pulmonary tissues were observed in the mice in the control group (Fig. 8c). As for the PCN group and *P. aeruginosa* group, we observed severe histopathological changes. Besides, those two groups also revealed a mass of infiltrating inflammatory cells, pulmonary traps, and pulmonary cell extinctions (Fig. 8c). In contrast, pulmonary histopathological tissues from LBP-treated groups showed moderate (Fig. 8d) and mild adverse changes (Fig. 8d). Normal spleen tissues were observed in control mice (Fig. 8d). As for the tissues in the PCN and *P. aeruginosa* groups, compared with the control group, the spleen’s normal tissue structure was lost. It was challenging to separate the red and white pulps. There was ample amyloid presence, while histopathological spleen tissues from the LBP-treated groups showed moderate and mild adverse changes (Fig. 8).

## Discussion

*Lycium barbarum* L. (L. barbarum) has been used in traditional Chinese medicine for a long time. LBPs are important active constituents of *L. barbarum*. However, there are limited reports concerning the effects of LBPs in fighting against bacterial infection. Previous studies have shown that *P. aeruginosa* has the ability to produce and secrete various virulence factors. The ability to cause increased resistance to commonly used antibiotics and the possession of numerous virulence factors render *P. aeruginosa* a challenging pathogen to treat in clinical settings. Thus, the need for new drug targets in *P. aeruginosa* is still high and requires animal models and testing of remedies with potential beneficial effects. Many monosaccharides and polysaccharides have been confirmed to protect cells from various environmental factors. In the present study, we found that LBP has direct benefits on *P. aeruginosa* infection.

Bronchial epithelial cell (tracheobronchial epithelial) has a cellular composition that prevents infection from inhaled particles. The ability of bacteria to adhere to mucosal epithelial cells, particularly concerning the respiratory system, is crucial to colonization and infection. In this study, our findings show that *P. aeruginosa* binds to populate related epithelial cell cultures rapidly. Moreover, *P. aeruginosa* cells were more uniformly distributed on the epithelial cell surface (Fig. 1g and h). As the dose of PCN concentration increased, the survival rate of epithelial cells decreased (Fig. 2a). Inhibitory effects and survival rate increased when the epithelial cells were pretreated with LBP at different concentrations (Fig. 2b). Soluble sugars stabilize the protoplast layer on the cell membrane surface and protect the intracellular enzyme protein. LBP is a mixture containing neutral polysaccharides, acid polysaccharides, and Hydrocarbon Acid (HA). Notably, Mannose receptors can recognize terminal mannose, *N*-acetylglucosamine, or fucose in polysaccharides in the cell membrane. The mannose-α-2/3-mannose structures are recognized by a surface lectin of cells, which mediate and complement antibody-independent phagocytosis, leading to the immunity system’s elevated protective function.

In this study, PCN-induced apoptosis emanates from two reasons. First, PCN contributed to the expression of pro-apoptotic protein caspase3 and Cyt-c and was elevated to decrease the anti-apoptotic protein Bcl-2 and Bcl-xl expressions. The regulatory mechanisms of apoptosis include but are not limited to caspases and BCL-2 family proteins. Caspase3 is the central molecule in apoptosis. Its activation is regulated by a series of signal transduction cascades, among which the interaction between antiapoptotic Bcl-2 and Bcl-xl proteins plays a vital role. The downregulation of Bcl-2 is reported to release cytochrome c from the mitochondria, resulting in caspase3 activation. Therefore, we speculate that PCN induces the expression of caspase3 by suppressing the expression of anti-apoptotic proteins Bcl-2 and Bcl-xl. Meanwhile, PCN could promote the expression of Cyt-c, which activates caspase9 and the downstream signal caspase3, resulting in the mitochondrial apoptotic cascade. As a result, the cleavage of caspase3 results in the occurrence of apoptosis. This is further substantiated by the data presented in Fig. 6. LBP inhibits or reduces the expression of apoptotic protein caspase3 and Cyt-c but increases antiapoptotic protein Bcl-2, limiting the exposure of epithelial cells to apoptosis. ROS generation is another hallmark event of apoptosis, and ROS level also elevates after PCN treatment. These lethal ROS further damaged the mitochondria, increased mitochondrial ROS generation, and increased the release of cytochrome c into the cytoplasm. Accumulation of mitochondria-derived ROS activates redox-sensitive transcription factors, facilitating anti-inflammatory or pro-inflammatory factors. ROS may promote the release of inflammatory cytokines, which may weaken antioxidant defenses and lead to oxidative stress. Following the PCN treatment, the release of large amounts of ROS disrupts the redox balance in cells and causes oxidative injury in mitochondrial function. PCN participates in ROS production, and the functional composition of LBP decreases intracellular ROS levels. Studies have shown that LBP is a mixture containing neutral polysaccharides, acid polysaccharides, and HA. The acidic polysaccharides show a larger free radical-scavenging activity than the neutral polysaccharides in LBP (9). This is related to galacturonic acid in the former and its ability to chelate metal ions and scavenge radically. Therefore, when cells are treated with PCN, the acidic polysaccharides in LBP neutralize the excessive ROS production caused by PCN through its unique free radical scavenging ability, thereby protecting cells from ROS damage.

Accumulation of mitochondrial-derived ROS activates redox-sensitive transcription factors, facilitating the production of anti-inflammatory or pro-inflammatory factors. They efficiently produce ROS and inflammatory cytokines, IL-1β, TNF, IL-6, and IL-8. Persistent inflammation can promote secondary tissue injury through the excess production of pro-inflammatory factors, such as TNF-α, IL-1β, and IL-6. The elevated levels of pro-inflammatory cytokines, which may cause immuno-mediated damage to the lungs and other organs, result in acute lung injury and, subsequently, multi-organ dysfunction. After the lungs and the draining lymph nodes, the spleen is the third organ involved in the immunity response generation. Therefore, histological analysis of vital organs is important to evaluate whether *P.A* or PCN could cause tissue damage, inflammation, or lesions. For *in vivo* study, *P.A* or PCN was systemically administered to mice. Our data show that P.A and PCN infection induced apoptosis protein caspase3 and Cyt-c expression and activation in mice lung and spleen tissues. Specifically, tissue damage is associated with the formation of inflammatory cell infiltration. Excess generation of pro-inflammatory cytokines can cause severe cellular and tissue injury by inducing oxidative damage of biological macromolecules and inflammatory cell infiltration. Cytokine(s) release from pulmonary tissue may trigger lymphocyte apoptosis in the spleen. Our study’s interesting finding is that LBP protects mice lung and spleen from *P.A*- and PCN-induced injury. Also, LBP could reduce the expression of caspase3, inhibit tissue injury in the lungs, and enhance the release of inflammatory cytokines in the spleen. These results suggest that LBP exerts protective effects *in vivo* and *in vitro*.

In this study, we investigate the effects of LBP on *Pseudomonas aeruginosa* infection-induced lung injury using air-liquid culture as an infection model. We confirm that *P. aeruginosa*-infected bronchial epithelial cells could lead to the occurrence of apoptosis caused by the overexpression of caspase3 in cells and imbalance of Bcl-2 family proteins. Meanwhile, the release of large amounts of ROS disrupts the redox balance in cells and causes oxidative injury in mitochondrial function. Excess generation of ROS and pro-inflammatory cytokines causes severe cellular and tissue injuries by inducing oxidative damage of biological macromolecules and inflammatory cell infiltration. Simultaneously, we confirm that LBP inhibits mouse bronchial epithelial cell apoptosis. Furthermore, LBP further inhibits the release of inflammatory cytokines and tissue injury by inhibiting ROS’ expression. Our research indicates the potential applicability of LBP as an antimicrobial agent.
